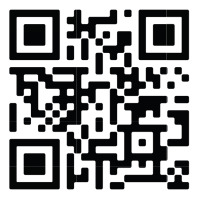# Unilateral pectineal suspension – A new surgical approach for apical correction of pelvic organ prolapse

**DOI:** 10.52054/FVVO.14.2.015

**Published:** 2022-07-01

**Authors:** D.I. Bolovis, C.V.M. Brucker

**Affiliations:** University Women’s Hospital, Paracelsus Medical University Nuremberg, Germany; Georg Simon Ohm Technical University Nuremberg, Germany

**Keywords:** Apical prolapse, lateral fixation, mesh-free, robotic surgery, uterus preservation

## Abstract

**Background and objectives:**

There are numerous vaginal and abdominal surgical approaches for the treatment of pelvic organ prolapse (POP). Even the standard techniques show great variability due to modifications depending on anatomy, available instruments and materials. Recently, the role of hysterectomy in prolapse surgery as well as the use of synthetic meshes have been questioned. Here, we present a standardised mesh-free minimally invasive pelvic floor reconstruction technique with uterus preservation.

**Materials and Methods:**

Unilateral pectineal suspension (UPS) is carried out in five defined steps with the use of the da Vinci Xi® surgical system. The desired anatomical result is simulated by intraoperative uterus manipulation. The cranial part of the pectineal ligament is used for lateral fixation. A non-absorbable suture is placed between the pectineal ligament and the anterior cervix to suspend the uterus in its natural anatomical position.

**Main outcome measures:**

For outcome measurement, degree of prolapse was assessed pre- and postoperatively according to the POP-Q system.

**Results:**

Unilateral pectineal suspension offers several advantages. Medial tension-free positioning of the uterus is achieved. The use of the cervix as fixation structure allows for excellent pelvic floor support and stable results. Normal pelvic floor mobility and natural axis of the vagina are restored.

**Conclusions:**

Unilateral pectineal suspension is an efficient minimal-invasive mesh-free procedure which allows uterus preservation and offers reliable level I support respecting the physiological pelvic anatomy. In addition, there is no need for ureteral dissection or bowel manipulation. The technique offers clinical standardization and can easily be integrated into the spectrum of modern surgical POP repair.

## Learning objective

The unilateral pectineal suspension (UPS) concept provides a mesh-free option for Level I correction preserving the uterus. It is a standardised and easily reproducible method due to the exact definition of the relevant surgical landmarks and the option for intra-operative simulation of the desired result. The suspension technique allows for preservation of the natural vaginal axis and physiologic pelvic floor mobility. The reduction of variability as provided by the standardised 5-step procedure shows a fast- learning curve and is easily adoptable for teaching and training purposes in every modern pelvic floor center.

## Introduction

Pelvic organ prolapse (POP) affects 30-50% of the female population depending on definition (Barber and Maher, 2013; Wu et al., 2009). Surgery is indicated in women with symptomatic POP who have failed or declined nonsurgical treatments. There are various vaginal and abdominal surgical approaches for the treatment of POP. As a first treatment, vaginal hysterectomy is still considered a standard of care in recent guidelines (NICE Guidance, 2019).

However, the role of hysterectomy in prolapse surgery has been increasingly questioned. Hysteropexy may be a viable alternative to hysterectomy in women with uterine prolapse. It can be performed either transvaginally by sacrospinous fixation, or abdominally or laparoscopically by placing a mesh or biologic graft form the cervix to the anterior longitudinal ligament, or by shortening of the uterosacral ligament. In addition, mesh- supported pectopexy can also be successfully performed preserving the uterus (Bolovis et al., 2021; Salman et al., 2022).

In recent years, the use of synthetic meshes for POP correction has become a matter of concern, leading to the classification of transvaginal mesh products as high-risk devices by the FDA in 2016 (Food and Drug Administration, 2016), followed by a complete ban of their production in 2019 (U.S. Food and Drug Administration, 2019).

Here we present UPS as a novel minimal invasive method for the operative treatment of apical POP fulfilling a broad range of quality criteria for POP correction. UPS provides anatomical reconstruction in a standardized 5-step procedure.

## Patients and Methods

The work presented was conducted at the maximum care University Women’s Hospital of Klinikum Nuremberg, Paracelsus Medical University, in accordance with the ethical standards of the Declaration of Helsinki. All patients were informed that a case description as well as documentation of their intraoperative situs might be used for publication purposes and gave their explicit consent.

Patients presented with apical prolapse of POP-Q 2 or higher as quantified according to the pelvic organ prolapse quantification system (POP-Q) described by ICS/IUGA (Haylen et al., 2016). Surgery was conducted in standard general anesthesia after informed consent had been obtained. The da Vinci Xi® surgical system was used with a three- arm reduced trocar setting as previously published (Bolovis et al., 2021). All surgeries were performed in Trendelenburg position under an intra-abdominal pressure of 12 mmHg. Postoperative ultrasound of bladder and kidneys was routinely performed on the first post-operative day to rule out urinary obstruction or retention.

The patients were examined vaginally two days postoperatively and during a follow-up visit scheduled three months after surgery to evaluate the outcome of their pelvic floor repair. Primary outcome parameters were defined as no recurrence of apical prolapse after three months according to the POP-Q criteria as specified above and subjective overall satisfaction with the status of the pelvic floor as assessed by a simple yes or no answer.

A set of quality parameters for POP surgery was defined and the intra- and postoperative results were compared to the proposed set.

## Results

UPS is performed in 5 defined steps. The uterus is intraoperatively guided to its desired position by two rotunda vulsellum forceps. The main anatomical landmarks for UPS are the pectineal (Cooper) ligament on the right side and the anterior cervix at the isthmo-cervical transition. The two structures are exposed (Figure 1a, 1b), followed by completion of the peritoneal dissection (Step 3, not shown). An ethibond #2 suture is placed through the pectineal ligament on the right at the S2 level, and subsequently placed into the anterior isthmo-cervical transition with three to four deep running parenchymal stitches. Adjustment of the correct position to achieve ideal midline uterine placement at physiologic height is controlled by vaginal examination. The suture is adjusted to keep the uterus suspended at the desired final position and tied (Figure 1c). The peritoneum is closed with a vicryl suture (Step 5). As a result of UPS, the uterus is positioned in midline anatomical position (Figure 1d), thereby restoring the natural vaginal axis and preserving physiologic pelvic floor mobility. The suspension procedure offers tension free adjustment, avoiding overcorrection.

**Figure 1a:**
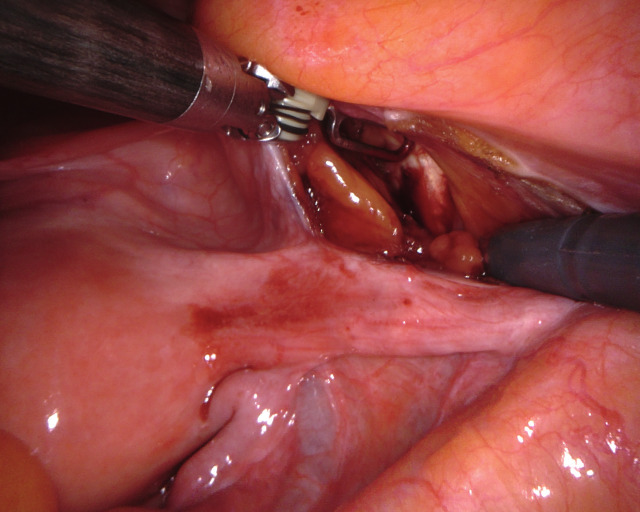
UPS operative technique: Step 1: The right pectineal ligament is exposed caudally of the M. psoas insertion at the S2 level.

**Figure 1b:**
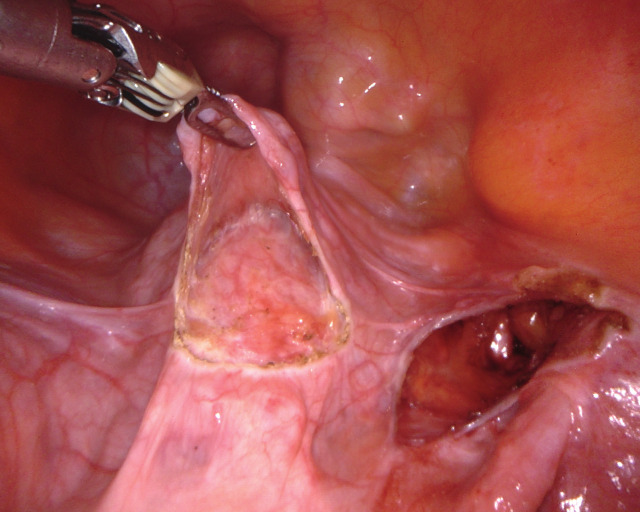
Step 2: A peritoneal bladder flap is prepared to expose the anterior cervix.

**Figure 1c:**
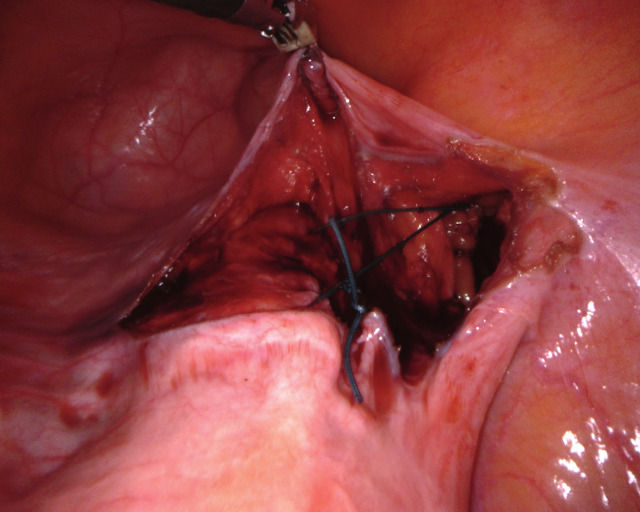
Step 4: A single non-absorbable suture is placed between anterior cervix and right pectineal ligament and adjusted to achieve the desired anatomical result.

**Figure 1d:**
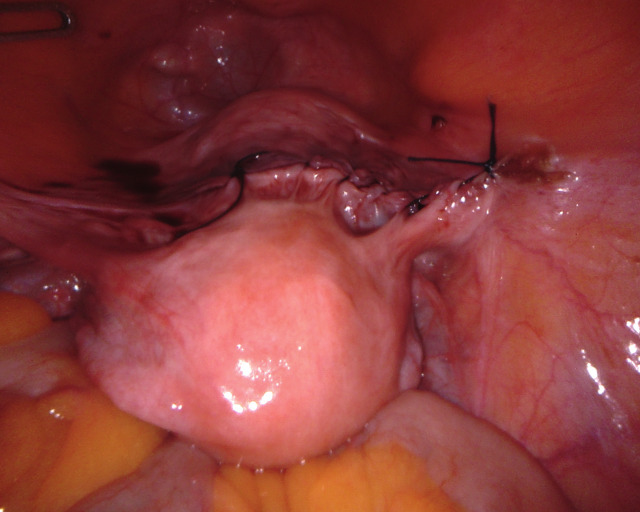
Step 5: Peritoneal closure is performed using vicryl #2.0 running suture, the uterus remains in midline physiologic position.

### Primary outcome parameters

POP-Q two days after surgery resembled the intraoperative result of POP-Q 0-1. During the follow-up examination three months after surgery the result of the apical fixation remained stable, and the patients confirmed their subjective overall satisfaction with their prolapse repair.

### UPS and POP quality criteria

We have compiled a set of intra- and perioperative as well as general quality criteria for POP correction (Table I). All postulated quality criteria are met by UPS.

**Table I:** Quality criteria for surgical correction of pelvic organ prolapse.

Intra- /perioperative quality criteria	General quality criteria
Anatomical POP correction	No subsequent dyspareunia
Preservation of vaginal axis	Stable result
Uterine preservation	MIS approach
Preservation of natural pelvic floor mobility	Mesh free
No vaginal tissue scarring	Fast procedure
Avoid bowel or ureter dissection	Day case surgery

## Discussion

We demonstrate UPS as a novel, minimally invasive, unilateral, mesh free suspension technique for isolated or combined apical POP correction in five defined steps. The procedure respects the physiological direction and angulation of the vaginal axis using an alternative fixation point. Reliable apical fixation is effectively accomplished in all POP stages, including stage 4 total prolapse. The uterus can be preserved unless uterine pathology warrants hysterectomy. The method provides midline positioning of the uterus and physiologic pelvic floor mobility. UPS can be combined with additional vaginal and abdominal procedures for the correction of POP and / or SUI during the same or a subsequent procedure. In summary, UPS fulfills a large panel of quality criteria for POP surgery, making it a strong candidate for a new standard of care.

The clinical justification for this technique is based on the combination of two thoroughly analysed principles: lateral suspension to the pectineal ligament and sacrospinous unilateral fixation of the uterus. As opposed to bilateral fixation we intentionally chose unilateral suspension since it avoids tension on the suspended structures and allows physiologic mobility. Respecting the direction of the round ligament for suspension a physiologic position of the suspended uterus is achieved. The use of the cervix as a stable fixation structure provides excellent pelvic floor support. The procedure avoids dissection along the ureter as well as bowel manipulation. Furthermore, it is assumed that the concomitant tension-free repositioning of the urethra due to restoration of the vaginal axis could avoid the appearance of de novo SUI.

The application of robotic technology offers a significant reduction of surgical compromise and adds value due to the simplification of suture and knot application. It facilitates anatomical dissection and minimises blood-loss, combining accuracy and surgical ergonomy. Nevertheless, the procedure can be standardised and performed by conventional laparoscopy to be broadly integrated into urogynaecology practice.

Extensive search of the international literature has shown that apical POP correction can be performed using various different approaches including sacrocolpopexy, vault suspensions, sacrospinous fixation and vaginal obliterative procedures ((NICE Guidance, 2019; ACOG Practice Bulletin, 2019; Geoffrion and Larouche, 2021). In recent guidelines and reviews, the use of the pectineal ligament as a fixation structure for POP repair has not yet been adopted. Nevertheless, pectineal ligament suspension has been successfully conducted in incontinence surgery (“Burch colposuspension”) for over 50 years and has been analysed thoroughly (Veit-Rubin et al., 2019; Sohlberg and Elliott, 2019).

In recent trials using pelvic MRI follow-up to compare postoperative results after various approaches for pelvic floor reconstruction, sacrocolpopexy (SCP) as well as sacrospinous fixation (SSF) have both been shown to deviate the vaginal axis in its medium and inferior portions (Juliato et al., 2019). As opposed to SCP and SSF, the vaginal axis was found to be near-normal in patients who underwent lateral mesh suspension (Pulatoğlu et al., 2021). Thus, using a lateral suspension point in the pelvis for prolapse correction at the level of S2 appears as a valid option for reconstructing the physiological axis of the vagina.

## Conclusions

In this study we describe the UPS technique as a novel approach for apical POP repair with intraoperative and immediate postoperative as well as short-term results. We are confident that this new method will find its place within the spectrum of recognised methods for apical POP correction due to its major advantages of being a standardised procedure without mesh application and the option to preserve the uterus. Certainly, intermediate and long-term outcomes have to be investigated in a larger cohort of patients. Retrospective as well as prospective studies are currently underway to address these issues, and long-term outcomes regarding the analysis of the sustainability of the anatomic results have to be awaited. We conclude that UPS fulfills a broad range of quality criteria for apical POP correction, making it a prime candidate for a new and innovative standard of care.

## Video scan (read QR)

https://vimeo.com/esge/review/687906690/1250f7db1d

**Figure:**